# Hymenoptera Genome Database: integrating genome annotations in HymenopteraMine

**DOI:** 10.1093/nar/gkv1208

**Published:** 2015-11-17

**Authors:** Christine G. Elsik, Aditi Tayal, Colin M. Diesh, Deepak R. Unni, Marianne L. Emery, Hung N. Nguyen, Darren E. Hagen

**Affiliations:** 1 Division of Animal Sciences, University of Missouri, Columbia, MO 65211, USA; 2 Division of Plant Sciences, University of Missouri, Columbia, MO 65211, USA; 3 MU Informatics Institute, University of Missouri, Columbia, MO 65211, USA

## Abstract

We report an update of the Hymenoptera Genome Database (HGD) (http://HymenopteraGenome.org), a model organism database for insect species of the order Hymenoptera (ants, bees and wasps). HGD maintains genomic data for 9 bee species, 10 ant species and 1 wasp, including the versions of genome and annotation data sets published by the genome sequencing consortiums and those provided by NCBI. A new data-mining warehouse, HymenopteraMine, based on the InterMine data warehousing system, integrates the genome data with data from external sources and facilitates cross-species analyses based on orthology. New genome browsers and annotation tools based on JBrowse/WebApollo provide easy genome navigation, and viewing of high throughput sequence data sets and can be used for collaborative genome annotation. All of the genomes and annotation data sets are combined into a single BLAST server that allows users to select and combine sequence data sets to search.

## INTRODUCTION

The insect order Hymenoptera—which includes bees, ants and wasps—is one of the most species-rich insect orders, with around 150 000 described species (1). Their diverse roles, such as pollinators of flowering plants (e.g. *Apis* and *Bombus*), parasitoids of other insects (e.g *Nasonia*) and cultivators of fungi (e.g *Atta* and *Acromyrmex*) make them vital to natural and agricultural ecosystems. For example, the value of crops in the United States attributed to pollination by the European honey bee, *Apis mellifera*, alone has been estimated at $16.4 billion (2). In addition to the economic benefits of crop pollination and natural pest control, hymenopteran insects have provided humans with products such as honey and beeswax for thousands of years. The order Hymenoptera also includes pests, such as fire ants (e.g *Solenopsis invicta*), which attack small mammals, and leaf cutting ants (e.g. *Atta* and *Acromyrmex*), which damage vegetation. Hymenopteran insects have been subjects of study not only because of their beneficial or detrimental impacts on agriculture and human welfare, but also because of their fascinating lifestyles. They have been used to address questions regarding the biology of social insects, symbiosis, parasitoidal relationships and invasive species. Ants, bees and wasps serve as premier models for investigating the evolution of eusociality, which refers to social systems in which many non-reproductive individuals provide support for the colony; eusociality has evolved independently in nine hymenopteran lineages (3). The first hymenopteran genome to be sequenced was that of *A. mellifera* in 2006 (4). Since then, genome sequences of over 20 hymenopteran species have been generated in projects that were aimed at addressing questions most pertinent to the subject species. The Hymenoptera Genome Database (HGD) (http://HymenopteraGenome.org), first reported in Nucleic Acids Research in 2011 (5), strives to make genomic data of hymenopteran insects easily accessible so that researchers can continue to leverage it to understand the biology of each organism, and to perform comparative studies to investigate processes that are common or diverse across the Hymenoptera order.

**Figure 1.**
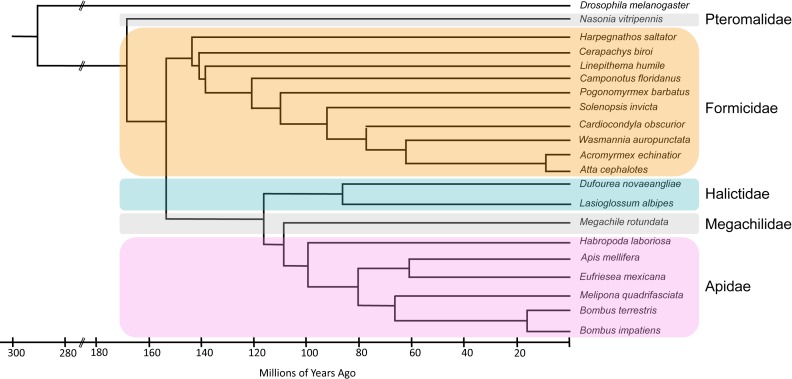
Phylogenetic relationships and approximate divergence times of species included in HGD. Families Apidae, Megachilidae and Halictidae are bees; Formicidae are ants; Pteromalidae are wasps. This figure was redrawn from (6,54–55).

HGD consists of three divisions, BeeBase, NasoniaBase and the Ant Genomes Portal, and covers a range of hymenopteran species that have diverged from a common ancestor approximately 170 million years ago (6), with the two most closely related species (the ants *Acromyrmex echinatior* and *Atta cephalotes*) having diverged as recently as 10 million years ago (Figure 1). Since the first report (5), the number of species represented in HGD has more than doubled (Table 1). The seven genomes included in the initial report were those of European honey bee (*A. mellifera*) (4), parasitoid jewel wasp (*Nasonia vitripennis*) (7) and five ant species – the leaf-cutter ant (*Atta cephalotes*) (8), the Florida carpenter ant (*Camponotus floridanus*) (9), the jumping ant (*Harpegnathos saltator*) (9), the Argentine ant (*Linepithema humile*) (10) and the red harvester ant (*Pogonomyrmex barbatus*) (11). In the past five years we have incorporated the genomes of 13 additional hymenopteran species. These are the Panamanian leaf cutter ant (*Acromyrmex echinatior*) (12), the ant *Cardiocondyla obscurior* (13), the clonal raider ant (*Cerapachys biroi*) (14), the red fire ant (*Solenopsis invicta*) (15), the little fire ant (*Wasmannia auropunctata*), the common eastern bumble bee (*Bombus impatiens*) (16), the buff-tailed bumble bee (*Bombus terrestris*) (16), alfalfa leafcutting bee (*Megachile rotundata*) (17), an orchid bee (*Eufriesa mexicana*) (17), a stingless bee (*Melipona quadrifasciata*) (17), the southeastern blueberry bee (*Habropoda labrosia*) (17) and two halictid bees (*Lasioglossum albipes* (18), *Dufourea novaeangliae* (17)). There are varying amounts and types of genomic resources for each species. The minimum set of data for each species is an assembled reference genome and at least one set of gene predictions, which may be a consortium gene set or an annotation release from NCBI. Our policy is to incorporate a genome after it has been published, unless we receive a request from an investigator prior to publication. Some species have additional information submitted by researchers, such as RNAseq alignments and genome re-sequencing alignments from variation studies. Since the initial publication, tools available at HGD have been used to support reannotation of *A. mellifera* (19), an ant genome comparative analysis (20), and community annotation of the two bumble bee genomes (16), *C. obscurior* (13) and *W. auropunctata*, which is not yet published.

**Table 1.** Species and data sets in HGD. JBrowse and BLAST is provided for all of the species listed

Species	New	NCBI Assembly Name^a^	HGD or Consortium Assembly Name	NCBI Annot. Release	OGS Name	Hymenop- teraMine	Ortho -DB	Ensembl Metazoa	Uni-Prot	Inter-Pro	GO	SRA	Pub-Med
Acromyrmex echinatior	√	Aech_3.9*	Aech_2.0	100	aech_OGSv3.8	√	√		√	√	√		
Apis mellifera		Amel_4.5	Amel_4.5	102	amel_OGSv3.2	√	√	√	√	√	√	√	√
Atta cephalotes		Attacep1.0*	Acep_1.0	100	acep_OGSv1.2	√	√	√	√	√	√		
Bombus impatiens	√	BIMP_2.0	Bimp_2.0	101	bimp_OGSv1.0	√	√		√	√	√		√
Bombus terrestris	√	Bter_1.0	Bter_1.0	101	NCBI Annotation	√	√		√	√	√		√
Camponotus floridanus		CamFlo_1.0*	Clfo_3.3	100	cflo_OGSv3.3	√	√		√	√	√		
Cardiocondyla obscurior	√		Cobs_1.4		cobs_OGSv1.4	√							
Cerapachys biroi	√	CerBir1.0*	Cbir.assembly.v3.0	100	armyant.OGS.V1.8.6								
Dufourea novaengliae	√		Dufourea_novaeangliae		Dufourea_novaeangliae_v1.1								
Eufriesa mexicana	√		Eufriesea_mexicana.v1.0		Eufriesea_mexicana_v1.1								
Habropoda labrosia	√		Habropoda_laboriosa.v1.0		Habropoda_laboriosa_v1.2								
Harpegnathos saltator		HarSal_1.0*	Hsal_3.3	100	hsal_OGSv3.3	√	√		√	√	√		
Lasioglossum albipes	√		Lalb_v2		Lalb_OGS_v5.42	√	√						
Linepithema humile		Lhum_UMD_V04*	Lhum_1.0	100	lhum_OGSv1.2	√	√		√	√			
Megachile rotundata	√	MROT_1.0	Mrot_1.0	101	Megachile_rotundata_v1.1								
Melipona quadrifasciata	√		v1.0		Melipona_quadrifasciata_v1.1								
Nasonia vitripennis		Nvit_2.1*	Nvit_1.0	101	OGSv2, nvit_OGSv1.2	√	√	√	√	√	√		√
Pogonomyrmex barbatus		Pbar_UMD_V03*	Pbar_1.0	100	pbar_OGSv1.2	√	√		√	√	√		
Solenopsis invicta	√	Si_gnG*	Sinv_1.0	100	sinv_OGS2.2.3	√	√	√	√	√	√		√
Wasmannia auropunctata	√	wasmannia.A_1.0*	Waur_1.0	100		√							

^a^An asterisk (*) next to an NCBI Assembly name indicates that the NCBI reference genome assembly differs from the consortium assembly, and HGD provides JBrowse for both assemblies.

## GENOME BROWSERS AND COMMUNITY ANNOTATION

A major focus of HGD has been community genome annotation. Since the previous HGD publication, we supported community annotation of the two *Bombus* genomes by computing homolog and RNAseq alignments, creating consensus gene sets, providing genome browsers (GBrowse (21)) with several gene prediction sets, and providing the Apollo annotation tool (22) with direct connections to Chado databases (23). More recently we replaced the HGD community annotation platform based on Apollo/Chado with WebApollo (24), a browser-based genome annotation tool that leverages the JBrowse (25) genome browser.

WebApollo has provided significant improvements over the previous Apollo/Chado annotation platform, both from data management and user perspectives. From the data management standpoint, loading data tracks, and collecting and exporting manual annotation data is simplified. From the user standpoint, WebApollo's integration with JBrowse allows fast genome navigation and viewing individual read alignments from high-throughput sequencing data sets. The user interface is highly configurable. Manual annotations are saved dynamically, so they are immediately available for viewing by other registered users. Gene model change histories are maintained; users can undo changes and administrators can view histories.

We have deployed WebApollo 2.0 for all species at HGD, providing publicly available JBrowse-based viewing of genome annotations. The WebApollo manual annotation tools and manual annotation data track are available to registered users for the species for which open annotation has been requested. We are actively annotating *A. mellifera* genes, and we provide tracks that allow users to focus on priority genes, such as loci that are in disagreement across gene prediction sets. For example, tracks include OGSv3.2 genes that are split and merged compared to RefSeq and the older gene set, OGSv1. RNAseq read alignment tracks are especially helpful for evaluating intron splice sites, or determining whether a gene should be split or merged. In addition to viewing tracks showing priority genes for annotation, users may navigate to genes of interest by performing a search with a protein homolog or cDNA sequence using the WebApollo built-in BLAT feature or the HGD BLAST server (described below).

## DATA MINING

We have developed a new data warehouse called HymenopteraMine using the InterMine data warehousing system (26). InterMine was originally developed for FlyMine (27), and now has become widely used for other model organism databases (e.g. (28–33)). The InterMOD consortium, a collaboration that includes the development teams for InterMine and five model organism databases, has worked to provide a platform for cross-species analyses through FlyMine, MouseMine, RatMine, ZebrafishMine, YeastMine and WormMine (34). The efforts of the InterMOD Consortium to ease the integration of genomic and functional data for cross-species comparison will prove to be especially valuable for research in non-model organisms, which relies heavily on comparative analyses. These efforts have made InterMine well suited to host the species of HGD. Integrating data across species in HymenopteraMine allows users to leverage cross-species data sets via orthologous relationships. The InterMine platform also eases the incorporation of external data sets, facilitating integration of the genome data in HGD with data from widely used insect genomic resources, such FlyBase (35), FlyMine (27), OrthoDB (36) and EnsemblMetazoa (37).

Another strength of the InterMine platform is its ability to resolve and manage multiple identifiers for the same entity. A common practice in hymenopteran genome projects has been for the research community to annotate the genome prior to annotation at NCBI. Members of the insect genome consortiums often perform their own automated gene prediction, followed by community manual annotation of gene families of particular interest, with analyses culminating in a consortium publication. Since their original publications, NCBI has annotated many of the hymenopteran genomes currently in HGD, resulting in the existence of at least two sources of genome annotations for these species, the consortium's official gene set (OGS) and the NCBI annotation release. HymenopteraMine addresses the common request among HGD users to provide tables with database cross-references or aliases for gene identifiers in different gene sets of the same species.

### HymenopteraMine data sources

Data in HymenopteraMine (Table 1) include genome assemblies and official gene sets developed by genome consortiums for the different species (8–13,15–16,18–19), annotations from NCBI RefSeq (38), Gene Ontology (39) and protein annotations from UniProt (40), protein family and domain assignments from InterPro (41), and *A. mellifera* gene expression information computed from RNAseq data downloaded from the NCBI Sequence Read Archive (SRA) (42). HymenopteraMine includes orthologues and paralogues from OrthoDB (36) and EnsemblMetazoa (37), facilitating comparison across the hymenopteran species and with *Drosophila melanogaster*. We have also acquired database cross references from NCBI for *A. mellifera* genes, and have computed database cross references for the other species by using IntersectBed (43) to identify overlapping gene predictions. Gene aliases for some of the species include the original gene prediction pipeline identifiers, such as those assigned by MAKER (44), or new versus old gene set identifiers, such as those for *A. mellifera* OGSv3.2 and OGSv1.0 (4,19). We used the gene2pubmed file downloaded from the NCBI Gene FTP site (ftp://ftp.ncbi.nlm.nih.gov/gene/DATA/gene2pubmed.gz) (45) to provide links to PubMed (46) abstracts, and used database cross references to link those abstracts to consortium OGS identifiers.

We have incorporated RNAseq data from the NCBI SRA into HymenopteraMine to allow users to investigate gene expression patterns. Our initial focus was to incorporate RNAseq data sets for *A. mellifera*, because at the time of download (September 2014) there were many more Illumina RNAseq data sets for *A. mellifera* than any other hymenopteran species in HGD (160 for *A. mellifera* compared to 31 or less for the other insects). We used the SRAInfo table for *Apis mellifera* to identify mRNA sequence data sets with 100 bp reads generated using an Illumina platform. We downloaded FASTQ files for 107 paired-end read runs and 51 single-end runs, trimmed for adaptors using Fastq-MCF (https://code.google.com/p/ea-utils/wiki/FastqMcf); we eliminated 17 paired-end runs and 10 single-end runs in which adaptors were not found. We then trimmed for quality using DynamicTrim (47). We aligned reads to the *A. mellifera* genome assembly Amel_4.5 using TopHat2 (48) and determined FPKM (Fragments Per Kilobase of transcript per Million mapped reads) and normalized read counts for each expression data set for transcripts in *A. mellifera* OGSv3.2 using cuffquant and cuffnorm, which are part the Cufflinks package (49). We also used CoverageBed (43) to determine raw read counts per transcript, and used the raw counts to compute RPKM (Reads Per Kilobase of transcript per Million mapped reads). HymenopteraMine users can retrieve expression levels along with metadata associated with expression experiments by entering OGSv3.2 gene identifiers, or they can retrieve gene lists based on gene expression constraints. More complex queries can use *A. mellifera* gene expression criteria and obtain data for orthologues, allowing researchers of hymenopteran species without comprehensive RNAseq data sets to leverage the *A. mellifera* expression resources.

### HymenopteraMine home page and quick search

The HymenopteraMine home page is accessible from the HGD navigation bar. HymenopteraMine has its own navigation bar that is available on all the HymenopteraMine pages. It provides a tab for the HymenopteraMine home page, a ‘Help’ tab linking to a tutorial, and tabs for the HymenopteraMine tools.

The HymenopteraMine home page provides a quick search tool and a quick list analysis tool, both of which accept multiple input data types. For example, a user can search for gene identifiers, transcript identifiers, gene symbols, functional annotation terms and species names. The quick search tool performs a full text search and supports wildcards; it returns results from all of the data sets loaded into HymenopteraMine and can be used to explore the data before performing more complex queries. Searching by species name with the quick search tool provides a list of all objects and data sets for that species. A faceted search tool in the quick search result page allows users to filter the results by category. The results of a quick list search can be used in downstream data mining.

In addition to quick search and list analysis forms, the home page provides tabs for the major data set categories in HymenopteraMine: ‘Genes’, ‘Gene Expression’, ‘Protein’, ‘Homology’ and ‘Function’. Each data category tab provides a list of relevant template queries. Clicking ‘More queries’ leads to a full list of templates on the ‘Templates’ page (described below), which is also accessible by clicking ‘Templates’ in the main HymenopteraMine navigation bar.

### HymenopteraMine reports

HymenopteraMine provides a report for each entity in the database. Each report is divided into sections including ‘Summary’, ‘Gene’, ‘Gene Expression’, ‘Protein’, ‘Function’, ‘Homology’, ‘Others’ or a subset of those categories, depending on the identifier searched. For example, searching a protein identifier would provide sections relevant to proteins. Each section of the report provides information in the form of a table that can be customized and downloaded in various formats. The Summary section of a Gene Report provides gene identifiers, symbols, description, organism, chromosome, strand and other identifiers such as aliases (identifiers from previous OGS versions) and database cross references. The Transcripts section provides information about the gene model (transcripts, exons, coding sequences) and gives a visual representation of the gene model highlighting the structure of the gene. Users can download FASTA-formatted sequences for each type of feature provided in the gene model section. Links are provided to JBrowse and to NCBI gene pages, when applicable. Transcript identifiers are linked to Transcript Reports, which include a Gene Expression section that provides raw read counts, normalized read counts, FPKM and RPKM values for RNAseq data with SRA metadata. The Protein section of the Gene Report includes protein name, accession and length. The protein name/accession numbers are linked to Protein Reports, with more information including protein family, GO annotations, InterPro domains, related publications, protein features, and curated notes from UniProt describing tissue specificity, function and developmental stages. The Function section provides GO annotation with evidence codes from the Biological Process, Molecular Function and Cellular Component ontologies. Clicking on the symbols to the right of each GO term leads to the directed acyclic graph showing all parents and children of the term, developed using the BioJS DAG Viewer (50). Clicking on a GO term provides a report for that term, a list of genes annotated with that term and tables showing relationships to other GO terms. The Homologue section lists homologues in other hymenopteran species and *Drosophila melanogaster*. The ‘Other’ section provides publications and a list of overlapping features.

### HymenopteraMine QueryBuilder

The QueryBuilder allows users to explore the underlying data model and construct queries that integrate data sources in HymenopteraMine. QueryBuilder does not require the user to have any programing experience, but it does require some practice. Investigating the predefined query Templates (described below) is a good way to get started. Users can navigate the hierarchical structure of data objects (classes) and subclasses by clicking on ‘Browse the Data Model’. Mousing over the symbol next to each class provides a description. The largest class is ‘Bio-Entity’, which is divided into ‘Protein’, ‘Protein Domain’ and ‘Sequence Feature’. The ‘Sequence Feature’ class is further divided into classes, such as ‘Gene’ and ‘Transcript’. Clicking on a class opens the Model Browser and reveals the class attributes, which can be selected to serve as search constraints or as output columns. For example the class ‘Transcript’ includes attributes ‘Description’, ‘Length’, ‘Name’, ‘DB identifier’, ‘Gene’, ‘Organism’. References between classes allow them to be combined within queries. Query construction is initiated by clicking the word ‘constrain’ next to a class. A box appears allowing the user to enter a constraint identifier; if no identifier is provided, all entities of the class will be searched. If the user is logged in and has already used the List tool (described below), an option will allow using the list to constrain multiple searches. Clicking on ‘show’ next to a class attribute adds the attribute as an output column. The Query Overview in the right panel shows the query construction. Once construction is complete, the ‘Fields selected for output’ section below the Model Browser can be used to rearrange column order. The query output can be filtered, sorted, reordered and downloaded in various formats, including XML, GFF3, tab-delimited text, JSON and BED. A detailed QueryBuilder example is provided in Supplementary Data.

### HymenopteraMine templates

The Templates page provides a list of easy-to-use pre-defined queries. The templates range in complexity, and can serve as learning tools to make more complex queries with QueryBuilder.

Some of the template queries were adopted from FlyMine (27), and others were custom developed for special use cases in HymenopteraMine. Clicking on a template provides a query interface that may include pull-down menus and may be pre-populated with an example identifier. Users may perform the query using the default identifier or may enter a different value. For some template queries, users are provided with constraint options that may include numerical operations, such as ‘less than’, ‘greater than’ and ‘not equal to’. Results are obtained by clicking the ‘Show Results’ button. Alternatively, clicking ‘Edit Query’ provides access to the QueryBuilder for query modification. As an example, QueryBuilder may be used to remove an identifier constraint, so that a query can be run on an entire data set at once. Modified templates can be saved for later use and can be exported in XML format to allow sharing among users.

### HymenopteraMine lists

The List tool allows users to create and manage lists of identifiers that can be used in further analysis. The user may enter a list of identifiers into the text box, upload a list as a text file or create a list by saving the results of a query. The pull-down menu in List indicates the objects for which identifiers may be entered. After the list is entered, the database performs a lookup of identifiers and prompts the user to disambiguate duplicate or unresolved identifiers. Logged-in users can save lists to refer to at a later date or to use in QueryBuilder. Before clicking ‘Save a list’, it is recommended that the user enter a name for the list so it can be easily recognized in later analyses. After a list has been saved, the QueryBuilder and Template queries automatically provide the option to use the list in any constraint with the same data type. For example, the template query ‘Gene->Alias’, which normally accepts a single gene identifier, will automatically include the option to input a saved list of gene identifiers created using the List tool.

### HymenopteraMine regions search

The Genomic Region search page allows users to perform an organism specific coordinate based search for genomic features. Users can paste a list of scaffold identifiers with coordinates into the text area, upload the list as a text file, or use the results of a query. For example, the output of the template query ‘All Gene → chromosomal location’ could be used in the Regions search after setting the sliding bar, to identity features within a specified distance of all genes.

## HGD BLAST USING SEQUENCE SERVER

We have deployed SequenceServer (http://sequenceserver.com) to create a unified BLAST interface that allows users to select and query multiple BLAST databases across the hymenopteran species. BLAST hits are linked to JBrowse viewers based on match coordinates when the search database is a genome assembly. When the search database is a CDS or peptide, BLAST hits are linked to JBrowse viewers based on the hit identifiers. The updated BLAST server is an improvement over the previous HGD BLAST interfaces, which used separate BLAST search pages for BeeBase, NasoniaBase and the Ant Genomes Portal, and did not allow the combination of individual search databases. SequenceServer also provides downloadable tab-delimited or BLAST XML reports and graphical overviews of the matches. The HGD BLAST server is similar to the BLAST server of the Fourmidable Ant Genome Database (51), but HGD provides data sets for a larger number of non-ant species. Fourmidable provides transcriptome assemblies for ant species that do not have genome assemblies, while HGD provides only genome assemblies and gene sets.

## GENOME ASSEMBLY AND GENE SET VERSIONS

For each species, the use of a single genome assembly and gene set by different researchers is advantageous, because it facilitates data sharing and comparison across studies. The existence of different assemblies for the same species is a common problem because genome assemblies and annotations are often updated when new data or improved assembly methods become available. Several of the genome assemblies in HGD have been updated. We strive to maintain the most recent assembly for each species and provide older assemblies in archive data set pages. For most of the ant genomes, the assemblies were submitted to NCBI after gene prediction and publication by the consortium. Processing and filtering for contaminants at NCBI has introduced changes to the assembly sequences. As a result, the annotation releases from NCBI and the respective consortium OGS (8–12,14–15) do not map to the same coordinate systems. For *Nasonia vitripennis*, the genome assembly has been through two upgrades at NCBI after the consortium publication (7), and there are two consortium official gene sets, neither of which was generated with the newest assembly. The existence of different gene sets and different assemblies is especially problematic when external data providers use different versions. For example, OrthoDB currently provides orthologs for each ant consortium OGS rather than the NCBI annotations on the latest assemblies; UniProt provides protein information for *Nasonia* OGSv1.2, while OrthoDB provides orthologues for *Nasonia* OGSv2.0, and neither of these gene sets is on the same coordinate system as the most recent NCBI annotation release. HGD attempts to address these conflicts in several ways. We provide multiple JBrowse/WebApollo instances for some species, with multiple assemblies and gene sets available for search using BLAST. HymenopteraMine provides coordinate information and links to JBrowse instances based on the more recent NCBI assemblies, but we also include functional information from external sources and database cross references for consortium gene sets. An ongoing effort at HGD is to migrate consortium gene sets to the updated assemblies.

## CITING HGD AND DATA SETS

Cite this article for the use HGD or its divisions (BeeBase, NasoniaBase and Ant Genomes Portal), tools such as HymenopteraMine, JBrowse/WebApollo and BLAST, or our code modifications available on GitHub (https://github.com/elsiklab/). In addition, cite the original genome publication for the use of a consortium genome or gene set downloaded from HGD. A list of genome publications may be found by clicking ‘Genome Consortium Publications’ in the HGD navigation bar.

## FUTURE DIRECTIONS AND CONCLUSIONS

The future goals of HGD are to add annotation resources for new hymenopteran genomes, improve connections with other insect bioinformatics resources, and further develop HymenopteraMine to enhance usability and incorporate new data types. We will continue to expand HGD with new species and additional high throughput sequencing data sets. We will strive to deploy WebApollo and BLAST resources for new genomes as they become available, rather than adhering to a predefined update schedule for those tools, and will rely on the NCBI genomes site, the Fourmidable Ant Genomics Database (51), PubMed searches and direct communication with researchers to identify new genome assemblies. We will continue to work with researchers to support community genome annotation, and encourage leaders of hymenopteran genome projects to contact us if they would like HGD to host community annotation prior to publication.

Our efforts in comparative genome analysis have been focused on either comparing hymenopteran species to each other or comparing hymenopteran species to model organisms, mainly *Drosophila*. We intend to improve connections to other model organism InterMine instances, and to improve connections to non-model insects via the i5k Workspace@NAL (52). Although our goal to support community annotation overlaps that of the i5k Workspace@NAL, the i5k Initiative is focused on ‘orphaned’ groups that do not have support for genome hosting (52,53). It was agreed early in i5k planning that HGD would continue to host the genomes of hymenopteran species. However, we have recognized the value of comparative insect genome analysis. To that end we are working with developers of the i5k Workspace@NAL, who will mirror HGD genome browsers and BLAST data sets allowing i5k users to compare Hymenoptera with other insects.

We will continue to modify HymenopteraMine to improve usability. Migrating annotations to upgraded assemblies, and computing gene aliases and database cross-references are perpetual efforts necessary for integrating data in HymenopteraMine. Therefore, we anticipate releasing HymenopteraMine updates on an annual basis rather than each time a new genome becomes available. We will continue to make source code modifications for HymenopteraMine and HGD BLAST available on GitHub (https://github.com/elsiklab/).

HymenopteraMine is the first InterMine instance for a group of non-model insects. With limited funding available for the study of non-model organisms, insect researchers need to leverage the knowledgebase and genomic resources of model species like *Drosophila melanogaster*. Similarly, researchers of hymenopteran insects need to leverage the resources created for the better-funded hymenopteran species such as *A. mellifera*. The InterMine platform accommodates these needs by enabling cross-species data mining using orthology. We have shown that InterMine can provide a solution to data integration and mining needs emerging with non-model insect genome sequencing initiatives such as i5k.
